# "Workhood"-a useful concept for the analysis of health workers' resources? an evaluation from Tanzania

**DOI:** 10.1186/1472-6963-12-55

**Published:** 2012-03-08

**Authors:** Karin Gross, Constanze Pfeiffer, Brigit Obrist

**Affiliations:** 1 Swiss Tropical and Public Health Institute, Basel, Switzerland; 2 University of Basel, Institute of Anthropology, Basel, Switzerland

## Abstract

**Background:**

International debates on improving health system performance and quality of care are strongly coined by systems thinking. There is a surprising lack of attention to the human (worker) elements. Although the central role of health workers within the health system has increasingly been acknowledged, there are hardly studies that analyze performance and quality of care from an individual perspective. Drawing on livelihood studies in health and sociological theory of capitals, this study develops and evaluates the new concept of workhood. As an analytical device the concept aims at understanding health workers' capacities to access resources (human, financial, physical, social, cultural and symbolic capital) and transfer them to the community from an individual perspective.

**Methods:**

Case studies were conducted in four Reproductive-and-Child-Health (RCH) clinics in the Kilombero Valley, south-eastern Tanzania, using different qualitative methods such as participant observation, informal discussions and in-depth interviews to explore the relevance of the different types of workhood resources for effective health service delivery. Health workers' ability to access these resources were investigated and factors facilitating or constraining access identified.

**Results:**

The study showed that lack of physical, human, cultural and financial capital constrained health workers' capacity to act. In particular, weak health infrastructure and health system failures led to the lack of sufficient drug and supply stocks and chronic staff shortages at the health facilities. However, health workers' capacity to mobilize social, cultural and symbolic capital played a significant role in their ability to overcome work related problems. Professional and non-professional social relationships were activated in order to access drug stocks and other supplies, transport and knowledge.

**Conclusions:**

By evaluating the workhood concept this study highlights the importance of understanding health worker performance by looking at their resources and capacities. Rather than blaming health workers for health system failures, applying a strength-based approach offers new insights into health workers' capacities and identifies entry points for target actions.

## Background

Quality of care strongly influences utilization of health care services and access to effective treatment. However, many studies from different countries of Sub-Saharan Africa have reported poor quality in terms of diagnostics and case management [1-5]. Other studies highlight the impact of health workers' bad attitudes towards their clients on quality of care [6-9]. Furthermore, health workers' discrimination and lack of respect towards the very poor and vulnerable is a theme that emerges in a number of studies [9-11]. Informal economic activities of health workers lead to exclusion, impoverishment and abuse of the poorest [12,13].

Shortages of health service inputs (trained staff, drugs and equipment) are facts in many health facilities in low income settings [12]. However, due to a tendency of perceiving bad performance as a problem of human resource management, there has been little attention to what access to resources means to health workers as social actors [14]. Also Franco and colleagues [15] noted the 'surprising lack of attention to the human (worker) elements'. Some recent studies from Scotland and Australia on health professionals working in remote communities provided evidence that health workers' access to resources is pertinent for their capacity to contribute to the social sustainability and health outcomes in their rural communities [16-18]. Drawing on Bourdieu's theory of capitals, Farmer et al. [17] showed that in particular social and cultural capital plays a crucial role. Health workers employed by the health system and living within their community, obtain an intimate understanding of the local culture and have networks inside and outside the community. As "boundary crossers" they are in an ideal position to operate in and across different fields, including health [18].

**Figure 1:**
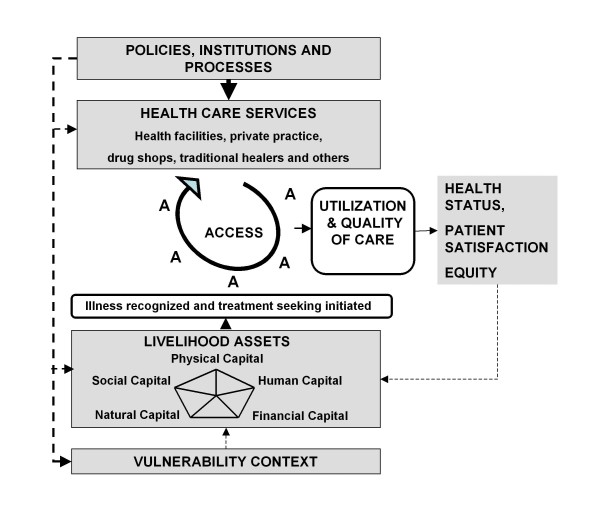
**Health Access Livelihood Framework**. Source: Obrist et al. [20].

Innovative livelihood studies have recently been applied to study how people mobilize livelihood assets (human capital, social capital, physical capital, natural capital and financial capital, see Table 1) on the household and community level in order to cope with health risks and gain access to health care [19-21]. Drawing on the Sustainable Livelihoods Framework of the United Kingdom Department for International Development [22] Obrist et al. [20] developed the Health Access Livelihood Framework (Figure 1). While the Sustainable Livelihoods Framework has mainly been used in relation to agriculture, poverty and development the Health Access Livelihood Framework linked studies on treatment seeking and health services with livelihood approaches and shifted their focus on people's access to critical resources during treatment-seeking. Studies explored people's way of mobilizing, combining and transforming capitals on the household and community level in order to access treatment for malaria [19-21] and other health problems [23]. Social relationships with relatives, neighbours or friends might therefore be transformed into financial means needed to pay health costs or borrowing a bike for the transport of a sick person [19,21]. Emphasis is given to the actors' capacity to not only cope and adjust to adverse conditions, but actively and creatively search for options [21]. Access as the "ability to derive benefits" from a resource [[24]:153] is a key issue and has been stressed in the Health Access Livelihood Framework [20]. However access to resources is strongly influenced by broader structures (role of government, private sector and donors) and processes (organizational, institutional, policy and cultural factors) in society [20,22].

**Table 1:** Comparison of livelihood and workhood resources

	**Livelihood assets **[22]	Workhood assets
**Definition**	A livelihood comprises the capabilities, assets (including both material and social resources) and activities required for a means of living (1.1)	A workhood comprises the capabilities and assets (material, social and cognitive resources) and activities required to fulfill job requirements

**Human capital**	Knowledge, skills, ability to work, good health	Size of available work force willing and able to work

**Physical capital**	Basic infrastructure and production equipment and means (transport, buildings, water supply and sanitation, energy, information)	Basic infrastructure (buildings, transport, electricity, water and sanitation) and production equipment and means (supplies and drugs)

**Financial capital**	Regular inflows of money and stocks (savings, credits, remittances and pensions)	Regular inflows of money and savings through the collection of user-fees

**Natural capital**	Natural resource stocks (land, forest, marine/wild resources, water)	-

**Social capital**	Vertical and horizontal networks, membership in formalized groups, relationships of trust, reciprocity and exchange	Vertical and horizontal networks inside and outside the community and within the health facility leading to relationships of trust, reciprocity and exchange

**Cultural capital**	-	Everyday perceptions, knowledge, skills and professional degrees gained through socialization that find its expression in particular professional culture

**Symbolic capital**	-	Power-related resources such as prestige, reputation and recognition gained through the possession of other capitals (economic, social, cultural, human).

**Figure 2:**
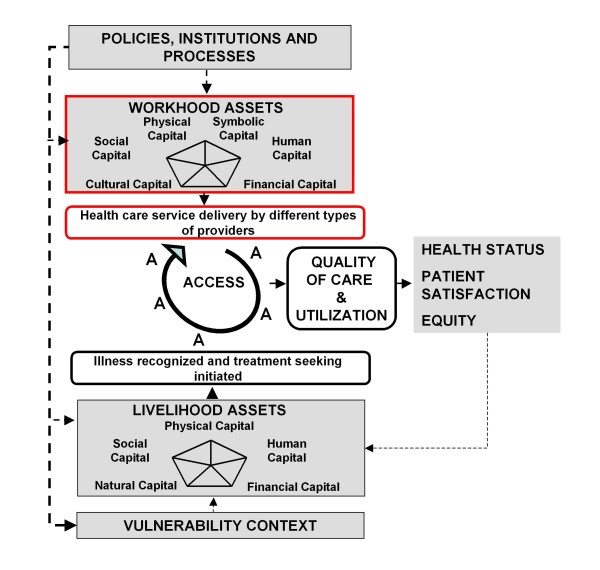
**Expanding the Health Access Livelihood Framework by workhood**. Adapted from the Health Access Livelihood Framework by Obrist et al. [20].

**Figure 3:**
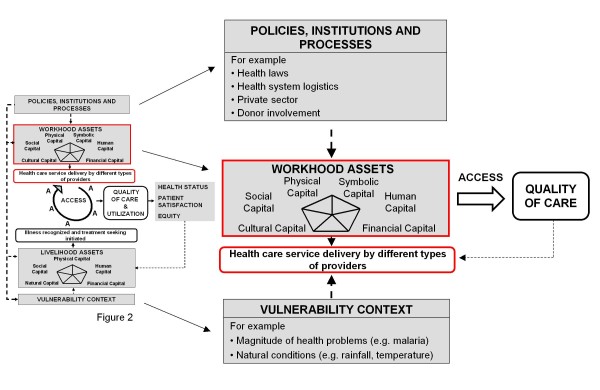
**The concept of workhood**.

Obrist and her colleagues understand social actors as the "potential driving force for improving access to effective and affordable health care" [20], at least on the level of those who seek care. However, they pay little attention to social actors delivering health services. Reflections on access to health care are rather coined by a health system perspective. By adapting the insights of the innovative livelihoods research to health workers' agency we like to expand the Health Access Livelihood Framework by the new concept of workhood (see Figure 2). By workhoods we mean the many forms of health workers' resources, capacities and activities required to provide effective health services to the community. By looking at workhoods the focus is laid on the individuals' actions in a given context rather than on their working environments or the health system. We understand health workers as social actors that are in a position to actively improve health and well-being of their clients and patients by mobilizing, combining and transforming assets. At the same time their agency is influenced and constrained by given structures [25] such as the local vulnerability context (e.g. magnitude of health problems) and policies (e.g. health laws), institutions (e.g. the private sector) and processes (e.g. health system logistics, see Figure 3). Health workers' access to workhood resources is a key issue for the provision of effective health services-especially in resource poor settings. Given that health workers must integrate their professional and private lives [14], we acknowledge their double need for livelihoods-to gain their own living-and workhoods-at work. Although we are aware of the potential interference, we shall focus on workhoods only.

This exploratory study aims at contributing new insights to the research of health workers' resources and capacities-a field that has not yet been investigated much-by pursuing three objectives. Firstly, it investigates the relevance of each type of workhood resource for effective health service delivery. Secondly, it explores health workers' ability to access these resources. Given that various structural and relational processes shape health workers' capacities to access resources, we analyze the means, processes and mechanisms that facilitate or constrain their access. Finally, the potential value of the workhood concept for future research is discussed. To do so, case studies were conducted in four rural Reproductive-and-Child-Health (RCH) clinics in south-eastern Tanzania using qualitative research methods. By health workers we refer here to trained nurses and untrained nurse assistants working in RCH clinics of government dispensaries and health centres (Table 2).

**Table 2:** Human and physical characteristics of the four selected health facilities

	D1	D2	HC1	HC2
Number of pregnant women who attended in 2008 according to HMIS data	625	366	554	1150

Distance to district hospital	47 km	20 km	27 km	64 km

Employed staff at dispensary/RCH clinic (health centre)	1 clinical officer 2 nurse attendants (1 nurse midwife had left for training)	1 clinical officer 1 nurse midwife (absent due to death in family) 1 nurse attendant	1 nurse midwife 1 nurse officer (absent due to illness) 1 Bwana Afya^a^	2 MCH Aids 1 Bwana Afya (absent)

Laboratory available	No	Yes (plans to upgrade dispensary to health centre)	Yes	Yes

Access to main road	No	Yes	Yes	Yes

Ambulance available	No	No	Yes (but under repair at the time of the study)	Yes

Note: ^a ^Health assistants based at the local health centre are locally known as *Bwana Afya *(literally 'Mr Health').

### Health workers and capitals-the concept of workhood

Drawing on the five core categories of capital used in the sustainable livelihood approach of the DfID [22] as well as on Bourdieu's conceptualizations of capitals, we suggest six categories of workhood assets in order to describe health workers' resources: human capital, financial capital, physical capital, social capital, and-instead of natural capital-cultural and symbolic capital (see Table 1). In the following, the possible relevance of each livelihood capital as a workhood asset and the inter-relationships between the various assets is discussed.

#### Human capital

In the Sustainable Livelihoods Framework, human capital represents the skills, knowledge, ability to labour and good health that enable people to pursue livelihood activities [22]. However, given that acquiring knowledge and skills requires social interactions and is shaped by class and culture specific socialization, we adhere to Bourdieu's conceptualization of skills and knowledge as cultural capital. Also, the fact that evidence based medicine is always adapted to local realities suggests that health workers' professional knowledge and skills are part of their cultural capital. Human capital as a workhood resource thus is defined here as the size of the available work force willing and able to work.

#### Physical capital

Physical capital comprises the basic infrastructure and the production equipment and means on which people draw in pursuit of livelihoods [22,26]. In health facilities, physical capital refers to buildings, transport, electricity, water and sanitation constituting the basis of a functioning infrastructure, and to drugs and supplies as production equipment necessary for the provision of effective health care. Most of these items are provided by the government, but might also derive from faith-based or other donor organizations. The acquirement of physical capital involves financial investments, logistics and planning of different scales and might require simultaneous skill- and capacity development as for example for new diagnostic and health technology.

#### Financial capital

Financial capital denotes regular inflows of money as well as stocks. Financial capital thus includes savings, credits, remittances and pensions that people use to achieve their livelihood objectives. It is probably the most versatile, but also the least available asset to the poor [22,26].

In the course of health sector reforms, many low- and middle-income countries have introduced user-fee systems at government health facilities as a means of mobilizing resources in order to increase the quality and coverage of health services. While salary payments build an important livelihood asset for health workers, financial workhood capital comprises of health facilities' regular inflows of money through the collection of user-fees and their accumulation on bank accounts.

#### Social capital

There are many debates what the term 'social capital' means. In the context of the Sustainable Livelihoods Framework, social capital has been understood as the social resources that are developed through vertical or horizontal networks, membership in formalized groups and relationships of trust, reciprocity and exchange [22]. Hence, social capital is not naturally given but is the result of continuous investment into relationships with other social actors and is bound to obligations, norms and values. Social capital thus constitutes "the ability to secure benefits through membership in networks and other social structures" [[27]:6]. On the other hand, it might be used to constrain opportunities of non-members, to restrict individual freedom and maintain power and status in a social hierarchy [28]. Given that health providers working in remote rural communities are deeply embedded in their rural communities they obtain social relationships of mutual trust, reciprocity and exchange within the community but also various vertical and horizontal networks within the health system [17,18,29].

#### Cultural capital

Drawing on Bourdieu's theory of capitals [30], we introduce cultural capital as a workhood resource. Bourdieu distinguishes three forms of cultural capital: incorporated, objectivized and institutionalized cultural capital [30,31]. Incorporated cultural capital comprises on the one hand (professional) skills and knowledge acquired through education and socialization, on the job-training, work experience and through the exchange of information with colleagues. Consequently, health workers are able to understand and use items of objectivized cultural capital belonging to their profession such as books, technical tools, guidelines that are not readily understandable and usable for lay persons. On the other hand cultural capital constitutes of everyday perceptions, skills and knowledge such as lifestyle preferences or the knowledge how to behave properly and interact with others. The incorporation of these resources through socialization is a life long process. Cultural capital becomes part of the individual human body and is not readily observable but rather finds its expression in particular professional cultures encompassing dispositions, values systems, habits, practices and knowledge that are tightly linked to their identity as health professionals [31,32]. Health workers' cultural capital therefore can be understood as a specific stock of knowledge [*Wissensvorrat] *in the sense of Schütz and Luckmann [33]. Of course, the "Wissensvorrat" of health workers is not exclusively built on experiences made during work, but interferes with experiences made as a private person. Finally, educational degrees, the institutionalized forms of cultural capital, procure health workers not only with access to the labor market (financial capital) and professional and non-professional networks (social capital) but also grants them symbolic capital in form of social status, prestige and power [31,34].

#### Symbolic capital

According to Bourdieu symbolic capital is "the form that the various species of capital (economic, social and cultural capital, inserted by KG) assume when they are perceived and recognized as legitimate" [[35]:17]. Symbolic capital encompasses power-related resources such as prestige, reputation and recognition and therefore influences not only actors' capacity to act but also their potential to access other resources [36]. Health workers working in remote communities usually obtain a considerable amount of symbolic capital due to their cultural but also social and economic capital. This is especially true for poor communities where educational levels are low and only few people obtain a degree in formal training and regular income.

## Methods

### The research setting

The Demographic Surveillance System (DSS) area in the Kilombero and Ulanga Districts in South-Eastern Tanzania served as study area. It comprises of 25 villages with an estimated total population of 92,000 in 2008 [37]. There is a wide mix of ethnic groups including WaNdamba, WaPogoro, WaBena, WaHehe and the newly arrived WaChagga and WaSukuma [21,38]. During the rainy season from December to April large parts of the Kilombero Valley are regularly flooded by the Kilombero River. This favours the cultivation of rice, which together with maize and cassava builds the main staple food and most important cash crop [20,38,39].

In the two districts a dense network of health facilities is available with dispensaries providing a basic range of curative and reproductive and child health (RCH) care to 6,000 to 10,000 people, health centres offering inpatient and higher level care to 50,000 people and hospitals serving as referral points for the facilities [40]. At the time of the study, a total of 10 first level (dispensaries) and two second level (health centres) health care facilities (9 public and 3 private) provided RCH care services on a weekly or daily basis from Monday to Friday and referred cases to two district hospitals. Since the early 1990s government facilities collect user fees. To reduce the impact of user fees on the most vulnerable groups exemption and waiver policies have been installed. Consequently, pregnant women and children under the age of 5 years are exempted from user fees [41].

### Data collection

The study was carried out between May 2008 and May 2009 as part of a wider research project [42]. Four government health facilities were selected in the research area for case studies: in each district the only available health centre and one additional dispensary. The selection of the dispensaries was based on the criteria of daily RCH service provision and a high number of pregnant women attending the RCH clinic based on patient registers. A more detailed description of the RCH clinics in terms of staffing, physical infrastructure and attendance numbers is provided in Table 2.

Case studies were conducted using different ethnographic methods: participant observation of daily RCH clinic procedures, informal conversation and semi-structured in-depth interviews with health workers. Out of eight health workers routinely offering RCH services at the selected health facilities, only five were present at the time of the study. Data collection was carried out at each health facility over a 1 week period in July 2008 by the leading researcher who is conversant in Swahili. She was supported by a local non-medical research assistant who could help with nuances of the language.

The method of case studies is valuable to enrich, validate and refine preliminary conceptual frameworks [43] and is particularly appropriate for the exploration of new topics [44]. The theoretical concept of workhood capitals was used as a "frame of reference". Participant observation and informal conversations with the health providers during and after work helped to understand clinic procedures and clarify questions on infrastructure and supplies, work procedures, health providers' responsibilities and patient-provider and provider-provider interaction. During the observations and informal conversations with health workers notes were taken to facilitate remembering activities and events, and were elaborated afterwards in descriptive field notes [45] in collaboration with the research assistant. The method of participant observation and health workers' high work load led to an involvement of the observers in administrative and registration work. Towards the end of the week, in-depth interviews were conducted with the five health workers available at the RCH clinics during the time of the study. A semi-structured questionnaire was used exploring health workers' training and position, their perceived work problems, their motivations, attitudes and work expectations, and their social relationships and interaction with patients, colleagues and supervisors. The in-depth interviews were tape-recorded after asking for health workers' permission.

### Data analysis

The in-depth interviews were transcribed and translated into English by two research assistants fluent in English and Kiswahili. The main researcher reviewed the transcript and original recordings and discussed ambiguities with the research assistants. Interview narratives and descriptive field notes were analyzed with MAXqda2 (VERBI Software, Marburg, Germany) using qualitative content analysis [46]. Analysis focused on observed events and health workers' narratives that were directly or indirectly related to health workers' lack or the mobilization and transfer of work resources in the context of structural forces. Text segments were identified and coded into capital categories. Within-case analysis was coupled with analysis across the four cases in search for patterns as suggested by Eisenhardt [44]. Contextual knowledge gained during 13 months of field work in health facilities and the communities helped to interpret the findings. Discussions with the RCH coordinators at the two districts in May 2008 and 2009 and reviews of national and international documents provided further background information for the interpretation and analysis of the collected data. Questions arising during the data analysis were addressed in follow-up and feedback visits at the four health facilities in April 2009. Thus, analysis was iterative and developmental as it served 1.) to refine and further develop the initial concept of workhood and 2.) to again evaluate the refined version with the data.

### Ethical considerations

The study was conducted within the frame of the ACCESS Programme which was cleared by the National Institution for Medical Research of Tanzania (NIMR/HQ/R.8c/Vol. I/66) [47]. Approval was further provided by the review boards of the Swiss Tropical and Public Health Institute (SwissTPH) and the Ifakara Health Institute (IHI). The study was authorized by the district RCH coordinators and the health facility staff granted permission to conduct the study at their facilities. All study participants provided oral or written informed consent after having been explained the purpose of the study and informed of their right to withdraw from the study at any time.

## Results

This section explores the relevance of each of the six workhood capitals (physical, human, financial, social, cultural and symbolic capital) for health providers' work. Two themes emerged from data analysis. The first theme describes health workers' experiences of working in a resource-poor setting. It illuminates what categories of workhood assets they were lacking in their daily work and provides health workers' explanations of why they failed to access them. The second theme focuses on how health workers overcame these challenges by mobilizing their own capacities.

### Lacking physical capital

Referring to the lack of physical capital, a nurse assistant of a dispensary situated in a remote village off the main road expressed her difficulties with organizing not only an ambulance, but transport in general:

*"We usually have a radio call to communicate with our colleagues over there. Often they tell us that unfortunately the ambulance is not available or that it is broken down. If I have an emergency case with a pregnant woman the issue of transport really is a problem. [In the village] over there, if a pregnant woman has a problem, firstly, there are cars available, and secondly, it is situated at the main road. They bring the patients to the main road and get easily transport, but here we get problems*" (Nurse assistant, D1).

The comment displays the twin problem of lacking physical capital the nurse has to deal with: on the one hand she can not rely on the health system, on the other hand general infrastructure such as transport means is weak. Moreover, in their daily work with pregnant women, health workers had to deal with the lack of work supplies, such as gloves and reagents needed for diagnostics, and drug shortages. Health workers attributed drug or supply shortages at the facility to either stock-outs at the district level or to the unreliability of the national drug supply system represented by MSD, the Tanzanian Medical Stores Department. Responsible for the procurement and storage of drugs, MSD is the unique supplier of drugs to government, faith-based and other non-governmental hospitals and health facilities (MSD 2010). Since the implementation of the Indent/Integrated Logistic System, MSD delivers drugs in customized kits according to individual health facility orders. However, health workers complained that MSD often does not supply the quantities ordered or fails to deliver certain drugs at all. Recent studies assessing the medicine supply system confirmed unconformities between the drug quantities ordered and delivered and reported long-term stock outs of essential drugs at MSD [48-50]. Moreover, discussions with the health workers and with the RCH coordinators revealed logistical problems at the district level where health workers are supposed to obtain specific items from the medical district store when they experience shortages at their health facility.

*"If we have shortage of drugs and other supplies we usually go to the district [capital] to request them. If they have them they provide us with the supplies but if they don't have them, then doctors tell us to come back another day. We return back to our facility for a while and then go again to the district" *(Nurse midwife, HC1).

### Insufficient financial capital

In order to enhance facilities' ability to improve their quality of care, in the early 1990s the government of Tanzania introduced user fees either in form of cost sharing or prepayment (i.e. Community Health Funds). Yet, buying drugs from other providers than MSD did not seem to be option for health workers. While one health worker argued that high prices discourage the purchase of drugs from private drug sellers, several other health workers considered the collected revenues to be insufficient to purchase drugs and supplies because of the money's use for other purposes.

*"Now we have this cost sharing, yes. If the situation is good, we can adjust ourselves at the health centre by buying these essential supplies which will help the society if money is available. If it is available, then, yes, this money has many uses. For example, those who cut the grass. There are watch-men employed that are paid by the facility. There is a woman who cleans the facility rooms, she is also paid. In reality it is not sufficient, only by squeezing squeezing [the money], yes, like that. We do not have electricity here, we use kerosene lamps [chemli]. This needs kerosene; it has to be bought every day. And as you know, the services of the children and pregnant women is for free, therefore those who are treated are adults, so the earning is not normal, that's the problem" *(MCH Aide, HC2).

Although health workers perceived exemptions to be the main cause for insufficient resources, evidence from the literature and the field suggests that low enrolment levels into cost recovery schemes, bad managerial practices and lack of transparency and accessibility hamper the potential for quality improvement [48,51]. Government health facilities often only have small shares of the revenues at their disposition and experienced administrative barriers to access money at the district bank account [48,52].

### Shortages of human capital

Staff shortages were experienced in all selected health facilities. A comparison with the national staffing level guidelines shows that dispensaries theoretically should be staffed with 5 people (2 clinical officers, 2 public health nurses and 1 nurse attendant) [53]. However, service provision in the two selected dispensaries was provided by 2-3 people (see Table 2). In both cases, assistant health workers who are minimally trained and often excluded from training courses due to the Ministry of Health's plan to phase them out (personal communication: RCH coordinator) solely provided the RCH services. Not surprisingly, health workers complained in the in-depth interviews about the lack of sufficient personnel and described their working situation as stressful.

*"If you would decide to stay a whole day at home [after delivering a baby during the night], there would be nobody here to do the work. Therefore, the nurse goes there (out-patient department) and returns here (RCH department) until she gets exhausted. Vaccines, children, pregnant women, patients, there is always someone"*. (Auxiliary nurse, D1).

In the health centres, the situation was better, but the staff still experienced a high level of workload and responsibilities due to absenteeism (see Table 2).

### Lacking access to cultural capital

Especially health workers with low education expressed their desire for cultural capital in form of better education. While other health workers were satisfied with the number of seminars they were able to attend in the last years, one health worker expressed frustration because her intention for further education was dismissed several times. In her explanations she referred to corrupt selection procedures in the past and her lack of social capital:

*"There at the district, in reality they chose especially the children of nurses. All of them went for studies. Now my colleague and me here, we come from the village. No wonder, my colleagues had many strategies, some even brought rice or maize, others they just know each other. We can go to the exams, you complete it, give it a try. No wonder, the name of your colleague was already there, they know who and who will go for studies, you fill it to give it a try" *(Nurse assistant, D2).

Lack of access to cultural capital was also highlighted in the case of another nurse assistant who was not allowed to perform HIV testing and counselling because she had not participated in the training seminar. Instead a nurse midwife had been sent to the seminar. However, in the meantime, the nurse midwife had left the facility and was transferred to the district capital illustrating the vicious circle of brain drain as access to cultural capital through institutionalized cultural capital (in form of degrees) raises the chances to be transferred to more convenient work settings.

### Social capital to overcome work problems

In-depth interviews revealed that health workers drawing on their own resources pursued a surprising array of strategies to cope with the problem of lacking drugs, supplies and human resources. Mobilizing social capital-and to a lower degree cultural and symbolic capital-played a significant role. In order to restock lacking drugs or supplies, professional relationships with the district authorities were activated either by the health workers themselves or through the doctor in charge. Yet, mobilization of social relationships with neighbouring health facility staff provided a more immediate strategy:

*"If we have shortages of [Sulphadoxine-Pyrimethamine (SP)], we use to go to request it [at the district] or we go to the neighbouring health facility. If we run out of [Sulphadoxine-Pyrimethamine (SP)], what do we do? We go to ask there" *(Nurse midwife, HC1).

Moreover, social capital was mobilized to access cultural capital. Health workers reported that they used to share information and knowledge gained in seminars and training courses with colleagues in staff meetings. Thus, knowledge was disseminated through peer education along the lines of professional networks within health facilities. However, health workers also mobilized social capital outside their professional networks in order to address lack of physical capital: In one health facility, social relationships with the drug sellers of a nearby shop had been established over time through regular purchases of gloves and other supplies, and in another one, the health worker relied on her social relationships with a resident logging company in order to compensate lacking ambulance and transport means in her remote village.

*"If the supplies are available in the nearby drug shop we run over to borrow them to get them without troubling people. Later on when the facility gets them they go to pay because we have a close relationship with the shop here. We buy small things there, apart from things like razors, gloves there, if we don't have them" *(MCH Aide, HC2).

*"If the ambulance is not available, we go to discuss it with the people from the Teak Company who are working here. If they have cars available, they help us. If there is no car, if the cars are used for work, it is a problem. But if there are vehicles they do help us, but not for free, the sick woman is supposed to hire the vehicle up to the hospital" *(Nurse assistant, D1).

### The role of symbolic and cultural capital

The fact that the nurse assistant was able to organize transport from people working for a South-African logging company, points not only to her social but also to her symbolic and cultural capital. After having worked for almost 10 years in the community, the nurse assistant obtained knowledge about potential resources and the necessary social relationships to act as a "boundary crosser" [18]. Her status as a health professional (symbolic capital) allowed her to interact with people from outside the community who might not be accessible for local people. The example highlights that in order to benefit from such opportunities not only social capital but also cultural capital in form of a thorough understanding of the system and the chances that it offers is important.

## Discussion

This paper sought to contribute to a field that has not yet been investigated much: health workers' access to work resources and their capacities to mobilize and transform them. Expanding the Health Access Livelihood Framework developed by Obrist et al. [20] this paper pursued three aims: firstly, to develop the concept of workhood to capture and explore health workers' resources and capacities, secondly, to illustrate and evaluate this theoretical concept with empirical data from four case studies, and thirdly, to discuss the usefulness and limits of the concept.

Drawing on the Sustainable Livelihoods Framework and on Bourdieu's conceptualizations of capitals we developed the workhood concept including six asset categories (Figure 3). Using the workhood concept as an analytical device we illustrated on the one hand what working in resource constraint settings means to health workers. On the other hand we showed that health workers have some capacities to mobilize and transform resources, thus enhancing their patients' health outcomes.

The case studies illustrated how structural constraints at multiple levels such as health system failures, weak infrastructure at the facilities and chronic shortages of trained staff led to the lack of physical, human, cultural and financial capital. Policies introduced to mitigate health system failures as for example user-fee schemes proved not to be functional. While the impact of lacking infrastructure, drugs and trained staff on health workers' performance and motivation has been stressed by several other studies [15,54,55], little attention has so far been given to health workers' capacities to access and mobilize work related resources from an individual perspective. Our study highlighted that health workers pursued a surprising array of workhood strategies in order to get hold of drugs and supplies (physical capital) and knowledge (cultural capital). Professional and non-professional relationships or networks were activated in order to organize needed resources such as drugs, knowledge and emergency transport. This points not only to health worker's social capital, but also to their symbolic capital-in form of status and recognition-and cultural capital-in form of knowledge about dealing with the civil service bureaucracy and other systems. Our findings support the arguments of a small working group around Farmer and Kilpatrick that in particular health workers' social and cultural capital is pertinent for their capacity to contribute to the social sustainability and health outcomes of their rural community. As Kilpatrick [18] argues health workers as "boundary crossers" are in an ideal position to operate in and across different fields. They benefit from the over-layering of skills, knowledge and perceptions coming from being a health professional, being a community member and from personal attributes. Thus, they are able to bridge structural holes and foster positive health outcomes [16-18,29].

By proving the usefulness of the workhood concept this study offers innovative and relevant insights into a field that has so far hardly been investigated and to earlier access studies in the health field:

Firstly, the concept of workhood constitutes a holistic analytical device that has the potential to inform and guide qualitative and participatory analyses of health worker performance. It shifts the attention away from health workers' performance as a problem of human resource management. Instead it helps identifying factors at different societal and health system levels that constrain or enhance health workers' access to workhood resources, thereby providing a understanding for what this means for health workers daily work experience.

Secondly, rather than blaming health workers for health system failures, with the strength-based approach inherent in the Sustainable Livelihoods Framework the application of the workhood concept opens new lines of inquiry. The study showed that health workers not only adapted to the constraining work setting, but obtained considerable capacities for mobilizing drugs, supplies or emergency transport when they were lacking them. The ability to not only cope and adapt but actively and creatively search for options and thus increase their competence in dealing with the constraint work setting is key for resilience building [36].

As the concept of workhood has been explored in a limited geographical setting and with a very small sample size, the generalization of these results is difficult. Thus, the value of the workhood concept needs to be further explored and tested in different geographical and institutional settings, and-more importantly-in combination with the livelihood concept. An expanded Health Access Livelihood Framework (see Figure 2) has the potential to better explain how people in interaction with individual health workers (rather than an anonymous health system) gain access to health care. However, health workers need to be understood as social actors whose performance not only depends on their access to resources but also on their own interests. What activities health workers adopt and the way they invest in resource mobilization and translation to clients is certainly driven in part by norms and values at the community and the health system level, but is also governed by their own (livelihood) priorities, attitudes and motivation. Ignoring health workers' own and their families' livelihood goals and activities, we left out an important factor influencing health workers' performance and the availability of work resources. Future studies should thus focus on the inference of workhoods with health workers' livelihoods.

## Conclusions

The study contributed to a small but increasing number of innovative livelihood studies focusing on access to health care and drew our attention to a new field: health workers' access to work resources and their capacities to mobilize and transfer them to the community.

Our study illustrates that applying the holistic and strength-based workhood concept to explore health workers' performance, allows for a better understanding of health workers' capacities to access and mobilize resources. The study showed that lack of physical, human, cultural and financial capital constrained health workers' ability for performance. At the same time they had learned how to cope with difficult working situations and pursued a surprising array of strategies in order to mobilize drugs, supplies, and transport means when resource shortages arise. Health worker's social, cultural and symbolic capital thereby played a significant role. The paper does not seek to romanticize the role of health professionals in rural areas, but intents to emphasize the need to explore health professionals' role in rural resource poor settings in a more holistic and in-depth way rather than blaming them for health system failures.

## Competing interests

The authors declare that they have no competing interests.

## Authors' contributions

KG was involved in the design and implementation of the study, field work, data management, analysis and interpretation of the data, and writing of the manuscript. BO and CP supported the design of the study and contributed to the manuscript. All authors have read and approved the final manuscript.

## Pre-publication history

The pre-publication history for this paper can be accessed here:

http://www.biomedcentral.com/1472-6963/12/55/prepub
